# Supplementary figures and images for: CRP Involved in Nile Tilapia (Oreochromis niloticus) against Bacterial Infection
Source: Biology (Basel). 2022 Jul 30;11(8):1149. doi: 10.3390/biology11081149 (PMC9405397; doi:10.3390/biology11081149)

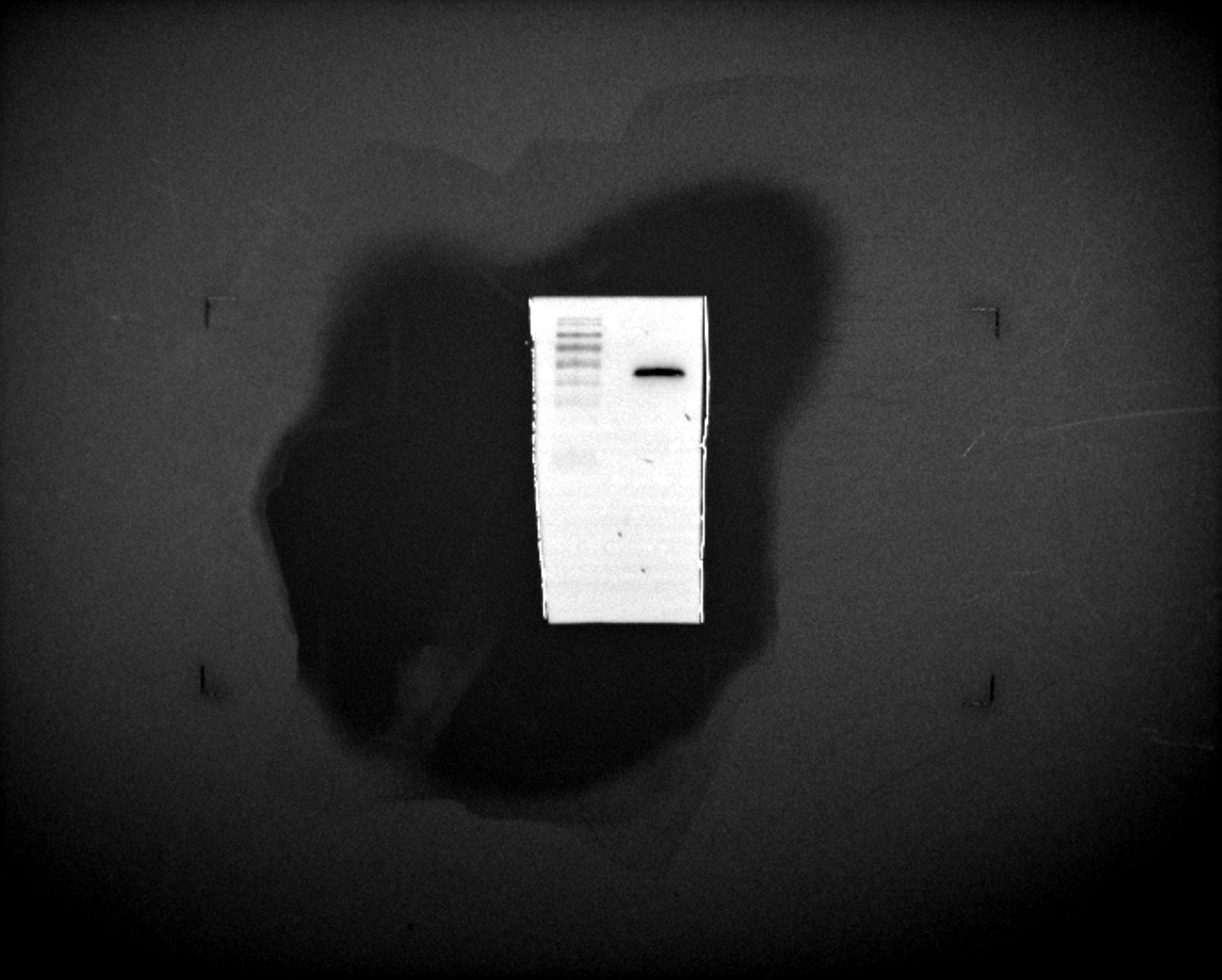

Supplement: Supplementary file 1 [file biology-11-01149-s001.zip › Supplementary Figure S1.tif]
